# Combined immunization with attenuated live influenza vaccine and chimeric pneumococcal recombinant protein improves the outcome of virus-bacterial infection in mice

**DOI:** 10.1371/journal.pone.0222148

**Published:** 2019-09-12

**Authors:** T. Kramskaya, G. Leontieva, Yu. Desheva, K. Grabovskaya, T. Gupalova, L. Rudenko, A. Suvorov

**Affiliations:** 1 Department of Molecular Microbiology, Institute of Experimental Medicine, Saint Petersburg, Russian Federation; 2 Department of Virology, Institute of Experimental Medicine, Saint Petersburg, Russian Federation; 3 Department of Fundamental Medicine and Medical Technologies, Faculty of Dentistry and Medical Technologies, Saint Petersburg State University, Saint Petersburg, Russian Federation; University of South Dakota, UNITED STATES

## Abstract

Influenza and its bacterial complications are a leading cause of morbidity and mortality worldwide. The effect of combined immunization with live influenza vaccine and recombinant chimeric pneumococcal protein in dual infection caused by influenza H1N1 and *S*. *pneumoniae* (serotype 3) has been studied. The combined vaccine consisted of the strain A/California/2009/38 (H1N1) pdm and chimeric recombinant protein PSPF composed of immunodominant fragments of the surface virulence factors of *S*. *pneumoniae—*PsaA, PspA, and Shr1875—associated with modified salmonella flagellin. Vaccinated mice were infected with the influenza virus 24 hours before or 24 hours after the onset of pneumococcal infection. The protective effect of combined vaccination was shown on both models of viral-bacterial infection.

## Introduction

Viral infections of the upper respiratory tract are often complicated by secondary bacterial infections [1, 2]. Influenza and its bacterial complications are the leading causes of morbidity and mortality worldwide. According to WHO, 250–500 thousand people die from influenza and its complications in developed countries each year [3]. There is experimental and clinical evidence that the influenza virus alters the host in a way that predisposes bacteria to enhanced adherence, invasion, and accumulation in the tissues [4]. In this regard, the prevention of both influenza and bacterial infections is very important. Associated virus-bacterial vaccination is a new way of preventing post-infectious bacterial complications. Previously, we proved the effectiveness of mixed immunization against influenza and group B Streptococcus in alleviating the course of a virus-bacterial co-infection. A mixture of live influenza vaccine and several polypeptides, recombinant analogs of group B streptococcal surface proteins, was successfully used as a vaccine preventing the GBS infection [5,6]. In the present work we employed a mixture of live influenza vaccine and PSPF protein, a genetically engineered preparation against pneumococcal infection, for vaccination. This option was chosen because pneumococci are the most common etiologic agents of post-influenza complications [1]. The protective effect of pneumococcal chimera PSPF in pneumococcal infection in mice was shown by us earlier [7]. In this study, we evaluated the protective effect of combined vaccination in two models of co-infection. In the first case, influenza infection preceded pneumococcal infection, in the second case followed it.

## Materials and methods

### Ethics statement

All the animal experiments were carried out under the guidelines of the “Rules of Laboratory Practice” of the Ministry of Health of the Russian Federation N° 708. The study was approved by the Local Ethics Committee for Animal Care and Use at the Institute of Experimental Medicine, Saint-Petersburg, Russia (protocol № 3/17 of 30.11.2017). Non-terminal procedures were performed under ether anesthesia. To control viral and bacterial load in the lungs, animals were euthanized under ether anesthesia and cervical dislocation. The health status of the combined vaccine challenged mice was monitored and recorded once a day for ten days post last vaccination. No animal showed any signs of illness following vaccine strain infection. No animals died as a result of the vaccination procedures.

The health status of the infected mice was monitored and recorded twice a day for ten days post bacterial or viral superinfection. Animals were euthanized immediately by cervical dislocation after ether anesthesia if they displayed abnormal behaviors (desire to be alone), ruffled fur, reduced mobility, hunchbacked posture. All efforts were made to minimize the suffering of the animals. No animals died before they met euthanasia criteria.

### Viruses and vaccine preparations

A live influenza vaccine (LAIV) strain A/17/California/09/38 (H1N1) pdm was provided by the Virology Department Institute of Experimental Medicine collection of viruses. The A/South Africa/3626/13 (H1N1) pdm influenza virus was obtained from the National Institute for Biological Standards and Control (NIBSC, UK) repository. All viruses were propagated in 8–10 days old chicken embryos and stored at -70°C.

Chimeric recombinant *S*. *pneumoniae* PSPF protein was expressed in *E*.*coli* and purified as described earlier [7].

### Bacterial culture

*S*. *pneumoniae* clinical isolates serotype 3 strain 73 were obtained from the collection of the Research Institute of Pediatric Infections (St. Petersburg, Russia). Pneumococci were cultured in anaerobic conditions at 37°C for 18 hours in THB medium with 20% horse serum (Difco). The Columbia agar (HiMedia, India) with 5% defibrinated sheep blood (Ecolab-Diagnostica, Russia) and 10% horse serum (Biolot, Russia) was used as a solid medium for cultivation and counting of the bacterial number.

### Animals

The 8-to-10-week-old female outbred mice were provided by the laboratory-breeding nursery of the Russian Academy of Sciences (Rappolovo, Leningrad Region, Russia). Mice were housed in groups of twenty in 400x250x200 mm cages (Plastpolymer, Russia), maintained under standard conditions and given ten days to acclimate to the housing facility. All animals were fed autoclaved food and water ad libitum. The weight of animals at the start of the experiments was 20.0±2.0 grams (mean±SEM).

### Immunization of mice

**Fig 1:**
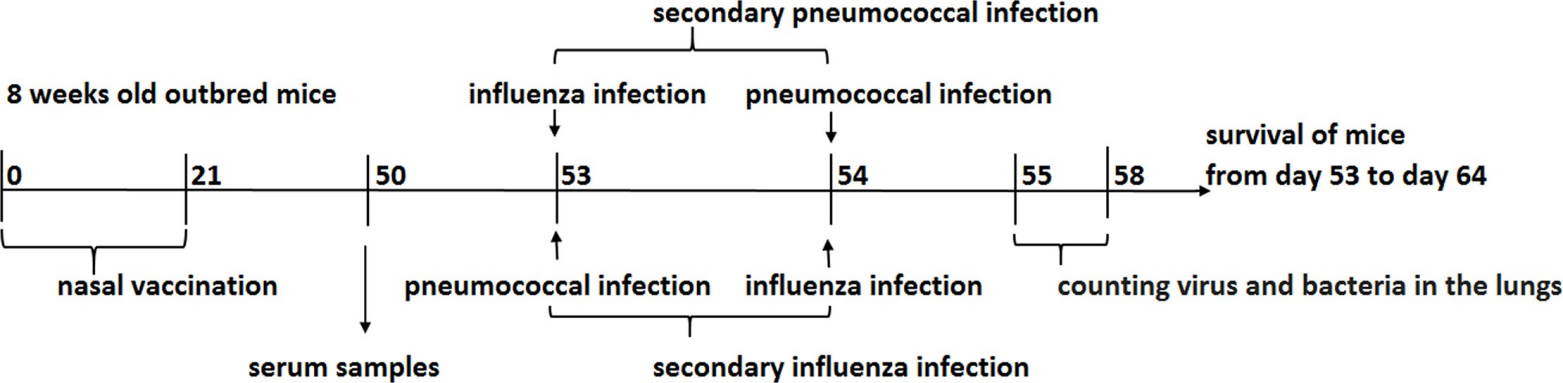
Immunization of mice and analysis of the protective efficacy of combined vaccination. General setup.

Randomly formed groups of mice (16 per group) were intranasally (i.n.) inoculated (Fig 1) with 50 μL of preparations as follows: 1) Diluted in PBS live influenza vaccine (LAIV) containing 6,0 lg 50% egg infectious dose (EID50) of the A/17/California /09/38 (H1N1) pdm vaccine virus; 2) PSPF vaccine containing the PSPF recombinant polypeptide diluted in PBS (20 μg); 3) mixed LAIV+PSPF vaccine; 4) PBS. Intranasal immunization was repeated on day 21 from the start of the experiment. Blood samples were taken from the submaxillary vein.

### Study of the protective efficacy of combined vaccination

In order to investigate the specific protective effectiveness of the immune response, mice were divided into two groups, and two variants of co-infection were induced on days 53 and 54 of the experiment (Fig 1). Secondary influenza infection: mice were nasally infected with sub lethal dose of *S*. *pneumoniae* serotype 3 (5 x 10^4^ CFU/50 μl), then challenged 24 hours later with sublethal dose of influenza virus A/South Africa/3626/13 (H1N1) pdm (3.5 lg EID_50_/50 μl). Secondary pneumococcal infection: mice were nasally infected with sublethal dose of influenza virus A/South Africa/3626/13 (H1N1) pdm (3.5 lg EID_50_/50 μl) and then challenged 24 hours later with sublethal dose of *S*. *pneumoniae* serotype 3 (5 x 10^4^ CFU/50 μl).

At the appropriate time, lungs were harvested and homogenized in PBS using a Retsch MM-400 ball vibratory mill. Serial 10-fold dilutions of homogenates were made in PBS and aliquots of the dilutions were plated on a dense nutrient medium, Columbia agar with 5% defibrinated sheep blood and 10% horse serum. Plates were incubated at 37° C in 5% CO2 for 14–16 hours before the colonies were counted under a microscope. The bacterial burden in CFU per organ was calculated and expressed as logarithm with base 10.

To determine the viral titer in the lung homogenates, the samples were homogenized in PBS containing 100 U/ml penicillin, 100 μg/ml streptomycin and centrifuged for 10 min at 6000g. The viral titers were calculated as EID_50_ using hemagglutination as the endpoint as described previously [8].

Survival rates were observed for 10 days after the start of the infection. The brains, lungs and spleens were removed from dead mice and put into contact with agar for a few seconds. The presence of bacteria was evidenced by the growth of typical mucous pneumococcal colonies within the area where tissue and agar came into contact.

### Specific immunoglobulin detection

Specific serum IgG levels were determined by ELISA in 96-well ELISA plates (Nunc) coated with the protein PSPF (2μg/ml) or A/New York/61/15 (H1N1) pdm (20 hemagglutination units (HAU) per 0.1 ml of the whole purified H1N1 virus) overnight at 4°C as previously described [7, 9].

The endpoint ELISA titers were expressed as the highest dilution that yielded an optical density at 450 nm (OD_450_) greater than the mean OD_450_ plus 3 standard deviations of negative control wells.

### Statistics

Data was processed using the Statistica software, version 6.0 (StatSoft, Inc. Tulsa, Oklahoma, USA). Means and standard deviations of the means (SD) were calculated to represent virus or bacterial titers. Serum titers of IgG were analyzed using log10 transformed data and presented as means±SEM. A log-rank test was performed for analysis of survival curves. We used a Mann-Whitney U-test to compare two independent groups. The p-values<0.05 were considered to be statistically significant.

## Results

### Immunogenicity of virus-bacterial vaccine components

The mice were vaccinated and processed in accordance with general experimental setup (Fig 1) and the protocol specified in the Materials and Methods section.

**Fig 2:**
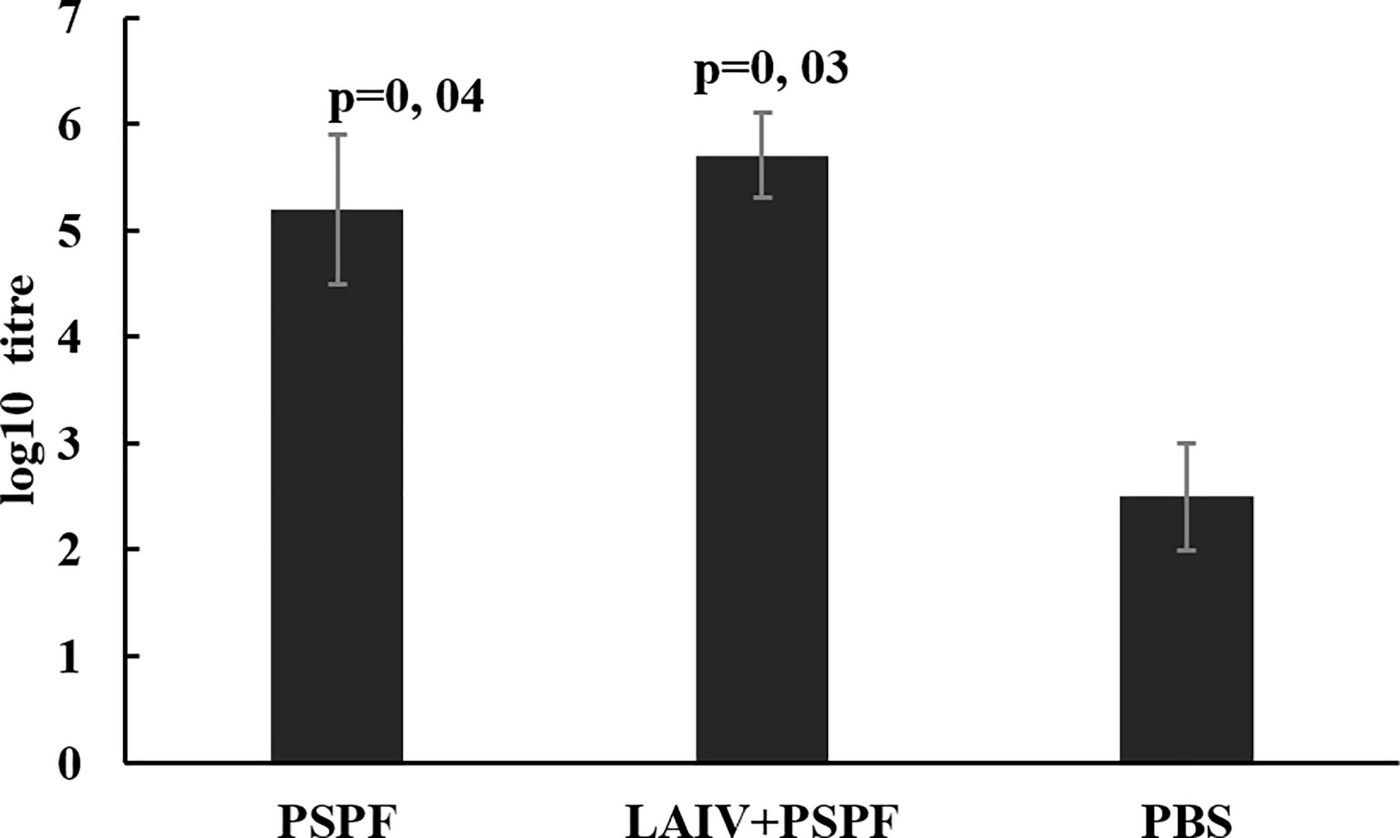
PSPF-specific serum IgG immune responses in mice after primary nasal vaccination and revaccination with PSPF or combined LAIV and PSPF vaccine. Reciprocal antibody titers (n = 8) were characterized as log10 (ELISA) and presented as mean±SEM; p-values provided are compared to mock-immunized animals (PBS).

**Fig 3:**
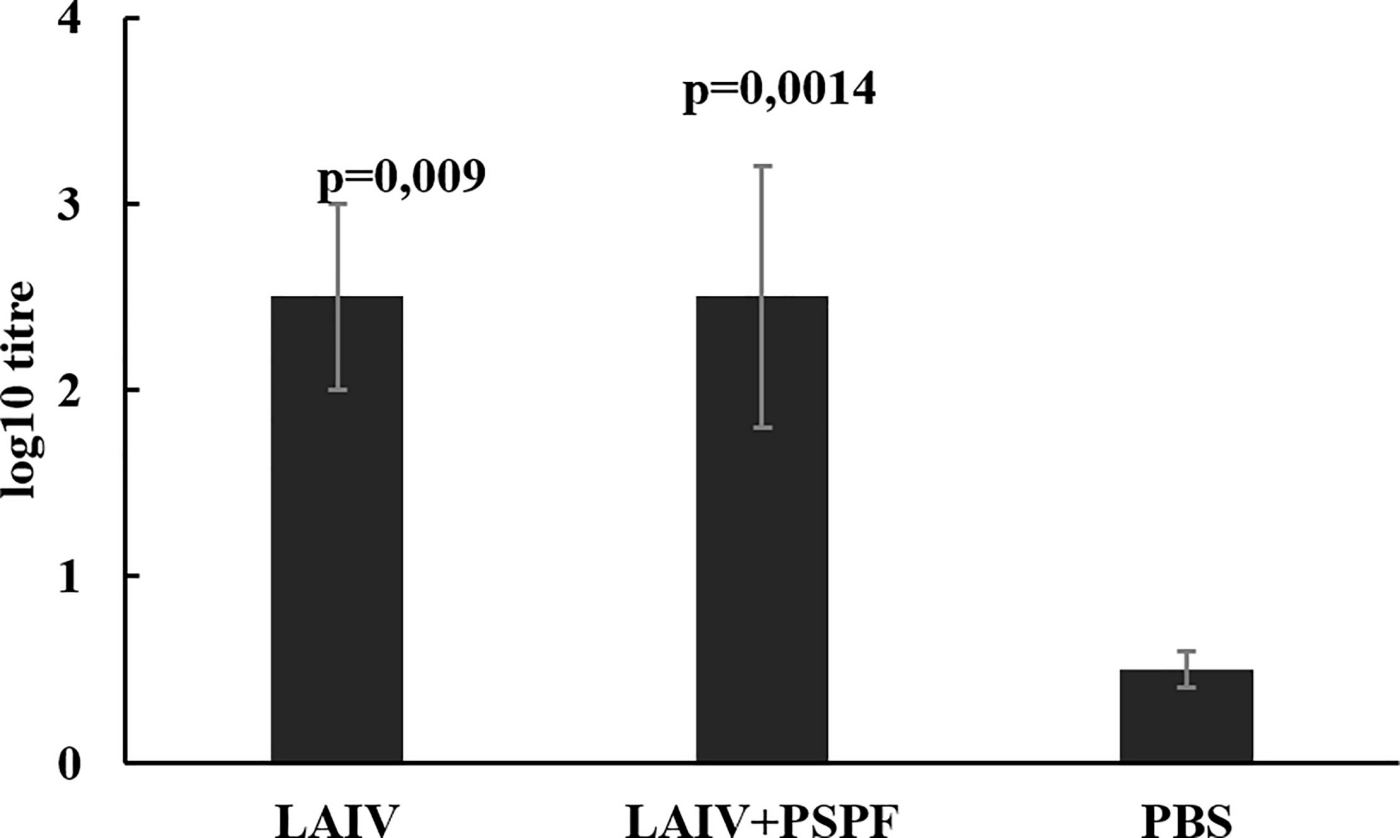
Virus-specific serum IgG immune responses in mice after primary vaccination and revaccination with LAIV or combined LAIV and PSPF vaccine. The whole purified A/New York/61/15 (H1N1) pdm virus was used for ELISA assay. Reciprocal antibody titers (n = 8) were expressed as log10 (ELISA) and presented as mean±SEM; p-values provided are compared to mock-immunized animals (PBS).

On day 50 of the associated vaccination course, the level of IgG antibodies specific to the chimeric PSPF protein and/or the H1N1 influenza virus in the mouse sera was measured. Both components proved to be immunogenic. Double intranasal vaccination with each of the two vaccine components and their mixture stimulated a comparable humoral IgG immune response (Figs 2 and 3).

### Protective efficiency of complex vaccination in the model of secondary pneumococcal infection

A mixed virus-bacterial infection was modeled by infecting vaccinated mice with a virus followed by bacterial challenge. Vaccinated mice were infected intranasally with influenza virus H1N1 strain A/South Africa/3626/13 (H1N1) pdm in a sublethal dose; the *S*. *pneumoniae* strain 73 of serotype 3 in a sublethal dose was administered intranasally 24 hours later.

In order to confirm the lethal synergism in the case of mixed virus-bacterial infection, we analyzed the severity of mono-viral and mono-bacterial infection in control mice. For these purposes, mock-treated mice were infected with the influenza virus or pneumococci only, and with the influenza virus and pneumococcus consecutively with an interval of 24 hours.

**Fig 4:**
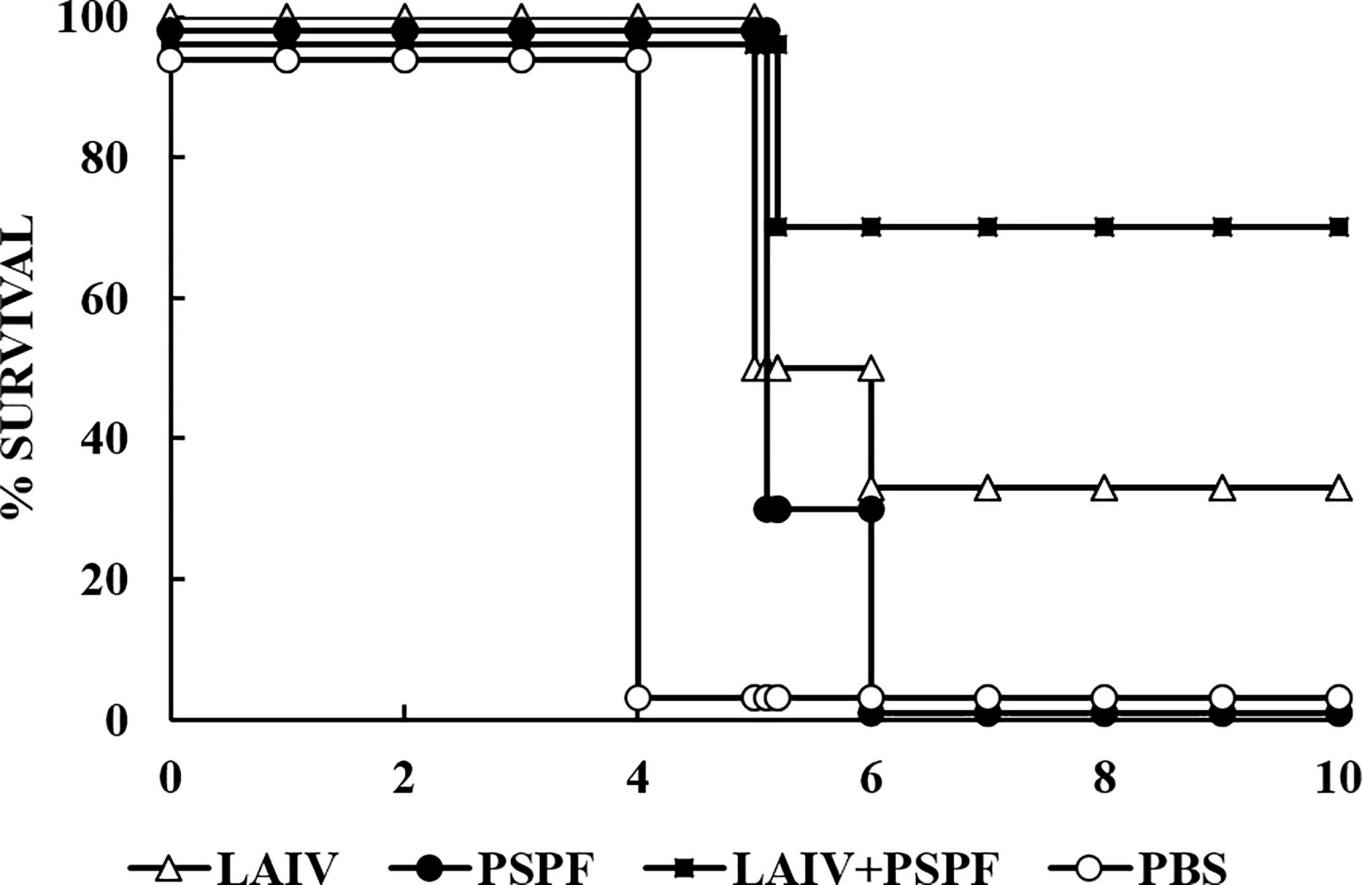
Vaccination protects mice from influenza complicated by secondary pneumococcal infection. Mice (n = 10 per group) were infected with sublethal dose of influenza virus H1N1, then challenged 24 hours later with a sublethal dose of *S*. *pneumoniae* strain 73 (type 3). The survival of mice was recorded daily for 10 days.

The severity of the infection was assessed by animal mortality rates within 10 days after the infection (Fig 4).

Viral and bacterial mono-infections did not cause the death of control mock-treated mice, which means that each pathogen separately was causing sublethal infections. At the same time, a co-infection of non-vaccinated mice with the influenza virus and pneumococci resulted in 100% death.

Immunization with PSPF protein and, accordingly, blood circulation of PSPF-specific IgG antibodies did not improve the clinical outcome of the dual infection. Mortality in this group of mice reached 100%.

Preventive vaccination with a single live influenza vaccine reduced mortality from 100% to 70%. The best result was obtained after vaccination with a complex virus-bacterial vaccine. This type of vaccine reduced the mortality rate to 30%. In both cases, this was a statistically significant improvement (p<0,05).

Simultaneously with mortality control, the viral and bacterial load in the lungs was analyzed. The lungs were recovered at 48 hours after the influenza infection; this corresponds to 24 hours of secondary bacterial superinfection.

**Fig 5:**
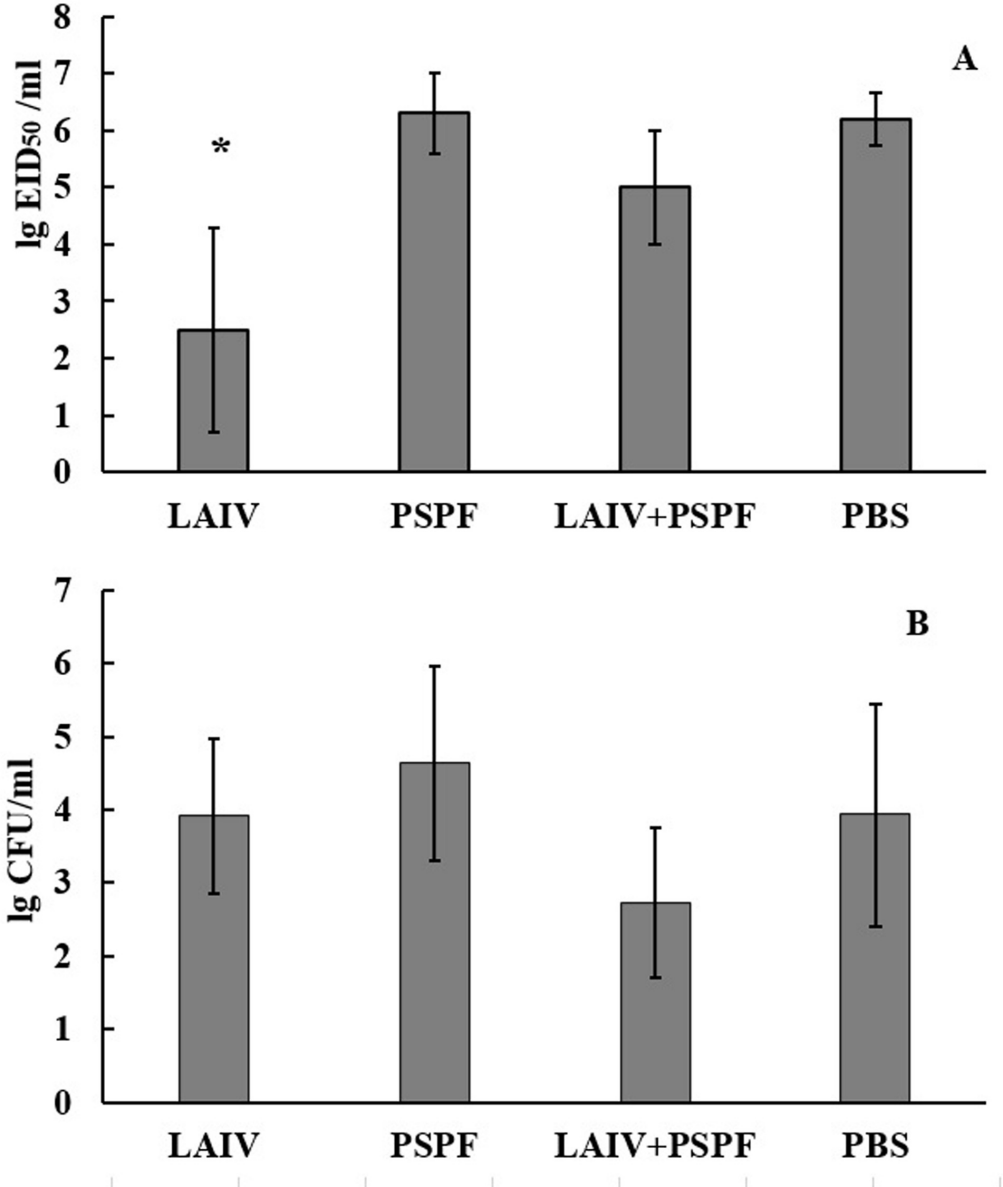
**Virus (A) and *S*. *pneumoniae* (B) isolation from the lungs after sequential challenge with influenza virus followed by *S*. *pneumoniae* type 3 infection.** Pathogens were isolated from lung homogenates (n = 6) obtained at 48 hours post viral infection. Bacterial and viral counts were characterized as log10 and presented as mean ± SD; *-significantly different values (p≤0.05) in comparison to mock-immunized animals (PBS).

After 48 hours, the influenza virus was isolated in the minimal titers in mice vaccinated with live influenza vaccine alone and in mixture with PSPF. In the PSPF and control groups, the viral burden in the lungs was maximal (Fig 5A).

The bacterial number was measured in the same lungs, in which the viral load was determined. Thus, at 24 hours from the secondary bacterial infection and, therefore, 48 hours after the primary viral infection, the smallest average number of pneumococci compared with the control was registered in the virus-bacterial vaccination group (Fig 5B and S1 Fig). In other groups, the average bacterial load on the lung was higher.

After monitoring the survival rate for 10 days after infection, we extracted the brains and lungs of the dead mice and made print cultures on to the blood agar. After incubating Columbia blood agar plates with 10% horse serum in 5% CO2 and at 37°C for 24 hours, typical mucous colonies of pneumococcus surrounded by a zone of alpha-hemolysis developed on the agar surface (S2 Fig). Thus, pneumococcus was present in the lung and brain tissues of all dead mice.

### Protective efficiency of complex vaccination in the model of secondary influenza superinfection

**Fig 6:**
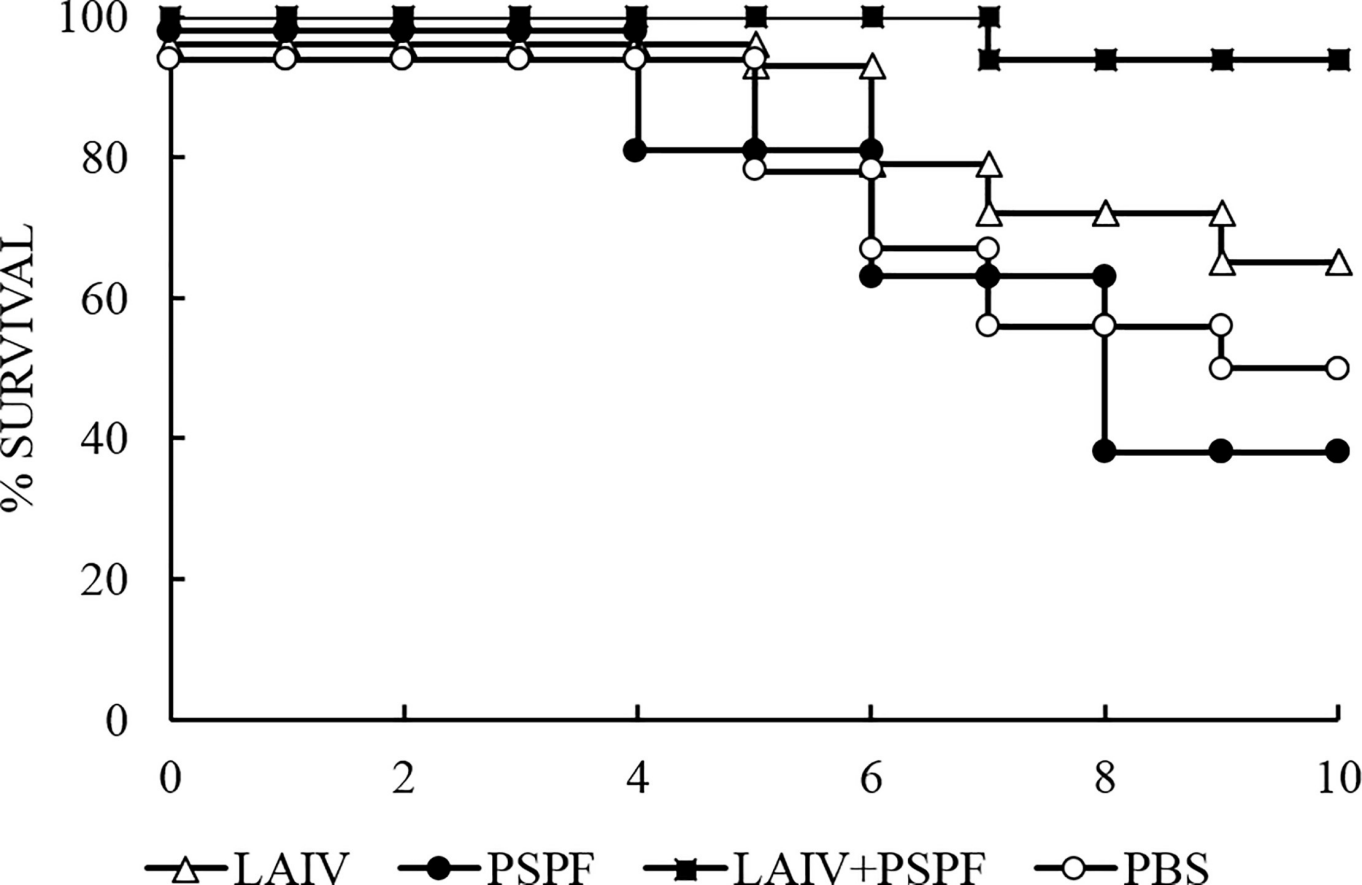
Vaccination protects mice from *S*. *pneumoniae* infection, complicated by secondary influenza infection. Mice (n = 14 per group) were infected with sublethal dose of *S*. *pneumoniae* strain 73 (type 3), then challenged 24 hours later with a sublethal dose of influenza virus H1N1. The survival of mice was recorded daily for 10 days.

Primary infection of vaccinated mice with a sublethal dose of *S*. *pneumoniae* strain 73 serotype 3 followed 24 hours later by challenge with influenza virus H1N1 in sublethal dose also increased the severity of the clinical course of infection (Fig 6).

The mortality rate of non-vaccinated mice after dual infection was 50%. Mono-viral and mono- pneumococcal infections did not cause death. Thus, in the absence of vaccination, the mortality of mice increased by 50% in the case when sublethal influenza infection followed the sublethal pneumococcal infection.

After prophylactic immunization with live influenza vaccine, mortality rates decreased from 50% to 35%. Vaccination with PSPF protein did not improve the clinical outcome of the co-infection. In this group, mortality of mice was 62%.

Statistically significant improvement (p<0,05) was noted after vaccination with a complex virus-bacterial vaccine. Mortality declined from 50 to 7%.

Thus, in both arrangements of dual infection, combined virus-bacterial vaccination provided statistically significant protection compared with the control and PSPF-vaccinated group, and a tendency to a decrease in mortality compared with vaccination against influenza alone was observed.

**Fig 7:**
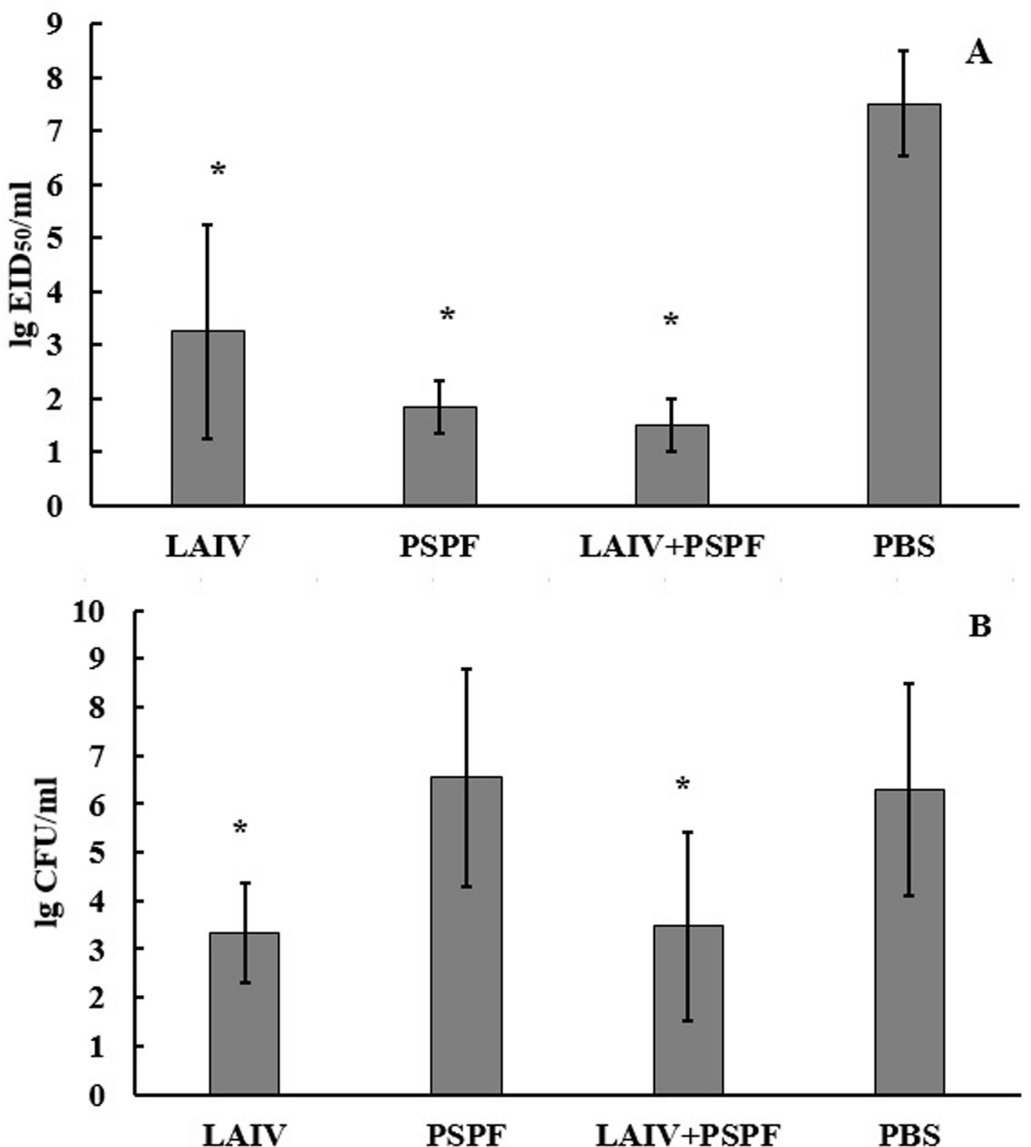
**Virus (A) and *S*. *pneumoniae* (B) isolation from mouse lungs after sequential challenge with *S*. *pneumoniae* type 3 followed by influenza viruses infection.** Pathogens were isolated from lung homogenates (n = 6) obtained at 48 hours post viral infection. Bacterial and viral counts were characterized as log10 and presented as mean ± SD; *- significantly different values (p≤0.05) in comparison to mock-immunized animals (PBS).

On hour 48 of the viral infection, the lungs were extracted to assess the intensity of the viral and pneumococcal infectious process. This period corresponded to 72 hours of bacterial infection. On hour 48, the maximum level of viral reproduction took place in the lungs of mock-vaccinated mice. A significant decrease in viral load relative to control was observed in mice vaccinated with live vaccine, in mice receiving antibacterial PSPF and their mixture (Fig 7A).

After 72 hours from the start of the bacterial infection the average bacterial load in the lungs in the PSPF group was not statistically different from the control group. Minimal accumulation of pneumococcus was noted in the lungs of mice vaccinated with live influenza vaccine and complex viral-bacterial vaccine (Fig 7B).

Counting of the bacterial number indicated that there are significant individual differences between experimental animals inside each experimental group. Mice differed sharply in the intensity of pneumococcal infection in lungs (S3 Fig).

Regularly registering mortality within 10 days after infection, we recovered the brain and lungs from the dead mice, placed the tissues on the medium of Columbia blood agar and then incubated imprints under favorable conditions for pneumococcal growth. Pneumococcus was detected in the tissues of all dead mice (S2 Fig).

## Discussion

The assumption that viral diseases provoke the attachment of bacterial infections was put forward in the XIX century and then repeatedly confirmed, especially in connection with the influenza pandemic of 1918 [10]. Many viruses can provoke a variety of bacterial superinfections. Viral-bacterial infections caused by influenza and *S*. *pneumoniae* are the most serious in their consequences. There are reasons to assume there is lethal synergism between these pathogens, due to which in the case of influenza, the attachment of secondary pneumococcal infections has especially severe clinical consequences [11].

The mechanisms of mixed virus-bacterial infections are still insufficiently understood, but are now extensively studied.

It is believed that influenza infection facilitates the colonization of mucous membranes with pneumococci. This can be due to direct damage to the epithelium of the bronchi and lungs [12–13], which is true for highly virulent strains of the influenza virus. Viral infection disrupts the functions of the ciliary epithelium on a variety of mucous membranes, and then removal of bacteria with mucus slows down [14–16]. Mild influenza infection changes the permeability of the alveoli, and swelling occurs due to the accumulation of serous exudate enriched with fibrin and inflammatory cells [17–18]. As a result, favorable conditions for the acceleration of bacteria growth arise. Pneumococcal adherence can be enhanced by the combined effect of both pathogens on the receptor apparatus of the epithelium. Several viral factors can increase bacterial adherence. Adhesion can be increased by the combined activity of viral and bacterial neuraminidases at the site of infection [19], stimulation of additional pneumococcal receptors by inflammatory mediators [20] or post-virus tissue regeneration by deposition of fibronectin, collagen and other matrix elements [21–22].

The development of lethal synergism is also determined by the specific influence of both pathogens on the innate immune system.

Both the influenza virus and pneumococci are recognized by pattern-recognition receptor (PRR), which triggers the synthesis and secretion of cytokines and immune cell immobilization. There is a significant similarity in the innate immune response to influenza and pneumococcal infections. A pro-inflammatory immune response to influenza and pneumococci is accompanied by the induction of IL-1, IL-6, TNF-, RANTES, MIP-1-alpha, IL-8, IL-10 and gamma interferon [23–25]. The overlap in the inflammatory mediators creates an opportunity for either interference with or augmentation of immune response during dual or sequential infection [4]. Due to synergistic stimulation of proinflammatory cytokines, an increased number of neutrophils and macrophages rushed to the site of the dual infection that caused substantial inflammatory damage, but did not contribute to bacterial clearance [26].

It is suggested that viral genome specifically signal the host immune system cells via TLR7 receptor molecules which results in reduced phagocytic capacity of macrophages for bacteria. It has been established that macrophages of TLR7-deficient mice destroyed a higher number of bacteria in influenza infection [27]. It was described also that influenza virus suppressed the immune response to systemic bacterial infection through the induction of glucocorticoids [28].

On the other hand, pneumococci can affect the virulence of the influenza virus and the severity of the inflammation process. Thus, bacterial proteases can increase the infectivity of the influenza virus by facilitating the HA0 cleavage process for the maturation of influenza virus progenies. Pneumolysin or pyruvate oxidase of pneumococci can directly stimulate inflammatory reactions [20].

It is obvious that the strategies for prevention and treatment of virus-bacterial infections must be based on the knowledge of the pathogenetic mechanisms of a co-infection.

Presently available experimental data cannot provide exact theories of pathogenesis of viral-bacterial infections. It is obvious only that the various microbial and immunological factors associated with viral and bacterial pathogens come into interaction multiplying the virulent potential of the both pathogens.

Modern methods of treatment of the viral-bacterial infections should include the prevention or treatment of influenza as a provoking factor in combination with the use of a variety of means of prevention of bacterial superinfection. It is shown, for example, that neuraminidase inhibitors used alone or in combination with antibiotics have a curative effect [19]. Vaccination against influenza reduces the incidence of secondary pneumococcal infections [29]. Combined vaccination against influenza and pneumococcal infections with 23-valent pneumococcal vaccine also positively affected the incidence rate in the period of influenza epidemics [30].

We propose a method for the simultaneous prevention of influenza and pneumococcal infection with a complex vaccine candidate containing licensed LAIV and a chimeric recombinant protein, which consists of fragments of several surface proteins—the virulence factors of *S*. *pneumoniae*.

This approach was successfully implemented by us on the model of a mixed virus-bacterial infection caused by the influenza viruses A (H7N3, H1N1, H7N9) and serotype II GBS. In these studies for prophylactic vaccination, we used live attenuated influenza vaccine strain A / 17 / Mallard / Netherlands / 00/95 (H7N3) in mixture with P6, ScaAB, ScpB1 and Stv recombinant GBS proteins. It was found that the complex preparation provides a higher level of protection against GBS infection than the antiviral and antibacterial mono vaccines. It is important that mixed vaccination with influenza A / Mallard / Netherlands / 12/00 (H7N3) wild type and ScaAB GBS protein provided an antiviral effect 5 days after vaccination [31].

In the present study, PSPF polypeptide was used as the antibacterial component of the vaccine. This is a recombinant protein composed of three immunodominant fragments of the surface virulence factors of *S*. *pneumoniae*: PsaA, PspA, and Shr1875 associated with flagellin.

Previously, we proved the protective efficacy of PSPF protein against infections caused by pneumococci of various serotypes [7]. PSPF protein was active in the form of free protein molecule under intranasal and subcutaneous administration [7, 32], as well as in association with a live pneumococcal vaccine based on the modified probiotic *E*. *faecium* in press. PSPF stimulated the accumulation of secretory IgA and systemic IgM and IgG antibodies, prevented bacteremia, providing an accelerated clearance of mice from pneumococcal infection.

In this study, a two-fold intranasal administration of a mono- and mixed vaccine stimulated a systemic IgG immune response to the influenza virus and the PSPF protein. The immunogenicity of the vaccine components in the mixture was not significantly different from their individual immunogenicity.

After vaccination, a dual infection was started by infecting mice with sublethal doses of influenza and pneumococcus in two different setups of infection: when viral infection preceded bacterial infection and vice versa.

In control mock-vaccinated mice, the application of secondary infection increased the severity of the outcome of the disease in both arrangements of the experiment. While primary mono infections were sublethal, bacterial superinfection resulted in 100%, viral—50% mortality in non-vaccinated mice group. The results were consistent with numerous observations of virus-bacterial synergism during influenza epidemics. The data of experimental studies of secondary bacterial pneumonia show that the outcome depended on the dose, the virulence of the infectious agents, and the time between infections [33]. In contrast to McCullers [19], who showed that the increase in lethality occurs only when bacterial superinfection follows influenza, and not vice versa, we registered a complication of the course of co-infection in both cases. In this study, the interval between infections was 24 hours. Previously, we studied the process of dual viral-bacterial infection, when the influenza virus was infected 3 days before or 3 days after the Group B streptococcus infection. In all cases, the aggravation of the disease and the transformation of a non-lethal form to a lethal infection were observed [34]. It is interesting to note that we registered the deterioration in survival also when mice were infected with influenza after stress, that is, the consequences of the non-infectious process similarly influenced the development of influenza superinfection. When mice were infected with a sublethal dose of the virus 24 hours after immobilization (for 6 hours), we observed the transition of a non-lethal infection to a lethal form of infection [35].

In this study, a complex vaccine seems to be the most effective means of preventing the consequences of viral and following bacterial infections. In the case of a secondary bacterial infection, mortality was reduced by 70%. After the secondary influenza superinfection, in the group of vaccinated mice mortality was 7%, but without vaccination, 50% of the animals died.

Vaccination with live influenza vaccine also slightly improved the course of the co-infection and reduced mortality by 30% and 15% in the case of secondary bacterial and influenza infection respectively. The data obtained coincide with clinical observations during epidemics regarding the positive impact of influenza vaccination on morbidity and mortality from secondary bacterial pneumonia [29]. The fact that the live vaccine had a positive effect on the course of mixed virus-bacterial infections is important in light of the fact that LAIV is widely used in several countries of the world including for vaccination of children [36].

Pre-vaccination with PSPF protein did not improve mouse survival after dual infection despite the circulation of bacterial-specific antibodies in the blood of immune animals. It should be noted that in primary monopneumococcal infection without viral complication the immune response to PSPF had a pronounced protective effect, as was shown earlier [4].

Previously we have shown that after intranasal administration of PSPF, both local specific IgA in the upper respiratory tract and the systemic IgG immune response were formed. In the present study, we analyzed only the level of PSPF-specific IgG in mice. In our case, the neutralization of pneumococcal infection with specific antibodies was not sufficient to change the clinical outcome of the viral-bacterial infection. Our results coincide with the observations, which showed no improvement in clinical manifestations of bacterial superinfection after antibiotic treatment [37].

Observation of bacterial and viral infections in the lungs revealed the greatest burden of both pathogens in the group of control animals. In two models, pre-vaccination with LAIV or LAIV + PSPF resulted in a significant reduction of the level of virus reproduction 48 hours after the onset of the viral infection. In the case when the viral infection followed bacterial infection, a decrease in viral reproduction with respect to the control was also observed in PSPF-vaccinated mice (Fig 7A). A decrease of virus multiplication in mice immune to PSPF can be explained by the release of active mediators, including interferons after the onset of bacterial infection in the immunocompetent mice [38]. In this case, the onset of viral superinfection could occur on anon- permissive background.

Along with a significant decrease in virus reproduction, a significant decrease in pneumococcal accumulation was noted. However, in the case when the influenza superinfection followed a bacterial infection, pneumococcal accumulation in the lungs did not differ from the control in PSPF-vaccinated mice (Fig 7B).

There are data showing that at the early stages of infection, pneumococcal burden in the lungs did not always directly correlate with the clinical outcome of the disease [39]. We found that in the group of mice vaccinated with PSPF 24 (Fig 5B) and 72 hours (Fig 7B) after the onset of bacterial infection, the bacteria accumulate as intensively as in the control. These results coincided with the high mortality rates in these groups (Figs 4 and 6).

Bacterial growth in the lungs and bacteremia likely lasted until the death of mice, which is confirmed by pneumococcal findings in the lungs and brains of dead animals (S1 Fig).

The outcome of this mouse model of mixed virus-bacterial infection depended on the spreading and replication in the tissues of both infectious agents. Immunity to influenza A (H1N1) virus (LAIV) slightly improved the outcome of a viral-bacterial infection. Vaccination with PSPF protein did not prevent the transformation of sublethal infection into a lethal infection, but the combined vaccination of LAIV and PSPF protein reduced mortality from the mixed virus-bacterial infection better than other vaccines.

Thus, in this study, we compared the protective effect of separate and combined usage of live influenza vaccine and recombinant chimeric pneumococcal PSPF protein against a virus-bacterial co-infection. It was shown that vaccination with a complex virus-bacterial vaccine effectively prevented the development of pneumococcal and influenza infections and drastically reduced the likelihood of the transformation of the primary sublethal infection into a lethal infection. Further research should provide an answer to the question whether the combination virus-bacterial vaccine prototype has the potential for preventing co-infections caused by the influenza A and *S*. *pneumoniae*.

## Conclusions

Currently the exact mechanisms of lethal synergism observed in co-infection of the influenza virus and *Streptococcal pneumoniae* remain unclear. In the present study, it was shown that vaccination of mice with a complex vaccine consisting of a live influenza vaccine in combination with the chimeric recombinant vaccine PSPF protein effectively prevents and dramatically reduces the transformation of primary sublethal infections to a lethal state. Detection of the protective effect of an attenuated virus along with an antibacterial vaccine protein can provide a new tool for the prevention of severe viral- bacterial infections.

## Supporting information

S1 Fig *S*. *pneumoniae* isolation from individual mouse lungs after sequential challenge with influenza viruses followed by *S*. *pneumoniae* type 3 infection. Pneumococci were isolated from lung homogenates obtained on 48 hours post primary viral infection (24 hours after secondary bacterial infection).
(TIF)

S2 Fig Brain imprint cultures on blood agar. Plates were cultivated for 24 hours at 37°C in an aerobic atmosphere enriched with 5% carbon dioxide. Colonies are surrounded by a zone of alpha-hemolysis. This is a typical example of *S*. *pneumoniae* colony growth on blood agar after contact with postmortem brain and lung specimens.
(TIF)

S3 Fig *S*. *pneumoniae* isolation from each individual mouse lungs after sequential challenge with *S*. *pneumoniae* type 3 followed by influenza virus infection. Pneumococci were isolated from lung homogenates obtained on 48 hours post-secondary viral infection (72 hours after primary bacterial infection).
(TIF)
