# Supplementary figures and images for: Combined immunization with attenuated live influenza vaccine and chimeric pneumococcal recombinant protein improves the outcome of virus-bacterial infection in mice
Source: PLoS One. 2019 Sep 12;14(9):e0222148. doi: 10.1371/journal.pone.0222148 (PMC6742370; doi:10.1371/journal.pone.0222148)

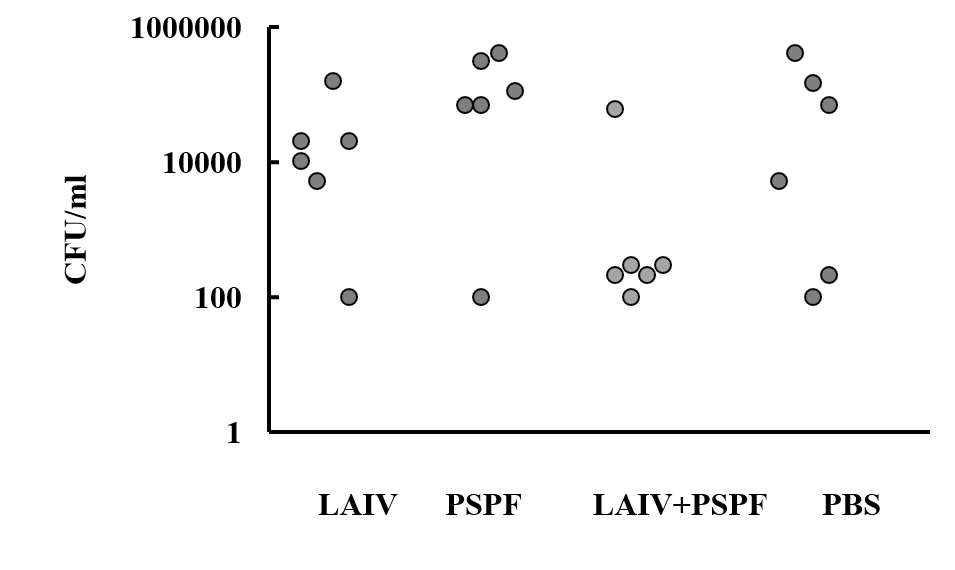

Supplement: S1 Fig — Pneumococci were isolated from lung homogenates obtained on 48 hours post primary viral infection (24 hours after secondary bacterial infection). (TIF) [file pone.0222148.s001.tif]

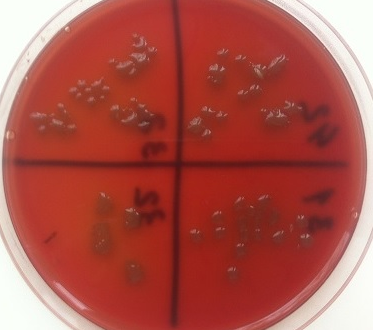

Supplement: S2 Fig — Plates were cultivated for 24 hours at 37°C in an aerobic atmosphere enriched with 5% carbon dioxide. Colonies are surrounded by a zone of alpha-hemolysis. This is a typical example of S. pneumoniae colony growth on blood agar after contact with postmortem brain and lung specimens. (TIF) [file pone.0222148.s002.tif]

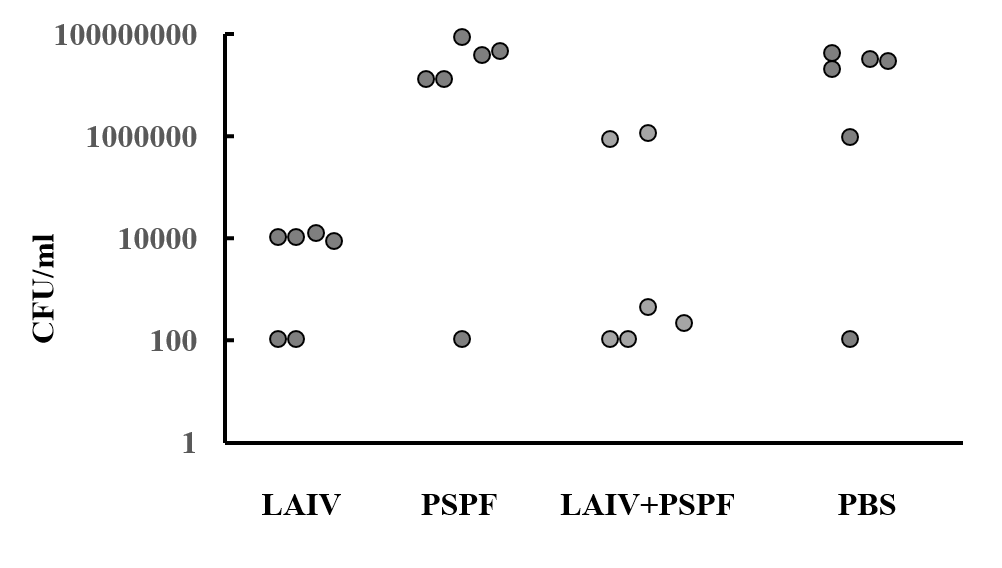

Supplement: S3 Fig — Pneumococci were isolated from lung homogenates obtained on 48 hours post-secondary viral infection (72 hours after primary bacterial infection). (TIF) [file pone.0222148.s003.tif]
